# Basic Performance Evaluation of a Radiation Survey Meter That Uses a Plastic-Scintillation Sensor

**DOI:** 10.3390/s24102973

**Published:** 2024-05-07

**Authors:** Keisuke Yamamoto, Ryota Shindo, Saya Ohno, Satoe Konta, Rio Isobe, Yohei Inaba, Masatoshi Suzuki, Yoshio Hosoi, Koichi Chida

**Affiliations:** 1 Course of Radiological Technology, Health Sciences, Tohoku University Graduate School of Medicine, 2-1 Seiryo, Aoba-ku, Sendai 980-8575, Japan; keisuke.yamamoto.r3@dc.tohoku.ac.jp (K.Y.); ryota.shindo.r5@dc.tohoku.ac.jp (R.S.); saya.ono.s8@dc.tohoku.ac.jp (S.O.); satoe.konta.q8@dc.tohoku.ac.jp (S.K.); rio.isobe.r2@dc.tohoku.ac.jp (R.I.); inabay@tohoku.ac.jp (Y.I.); masatoshi.suzuki.c7@tohoku.ac.jp (M.S.); 2 Department of Radiation Biology, Tohoku University Graduate School of Medicine, 2-1 Seiryo, Aoba-ku, Sendai 980-8574, Japan; hosoi@med.tohoku.ac.jp; 3 Department of Radiation Disaster Medicine, International Research Institute of Disaster Science, Tohoku University, 468-1 Aramaki Aza-Aoba, Aoba-ku, Sendai 980-0845, Japan

**Keywords:** Geiger–Müller (GM), radiation survey meter, plastic scintillator, radiation monitoring/measurement, plastic-scintillation survey meter, β-ray, Fukushima nuclear accident, environmental radiation, surface radiation contamination, electron beam

## Abstract

After the Fukushima nuclear power plant accident in 2011, many types of survey meters were used, including Geiger–Müller (GM) survey meters, which have long been used to measure β-rays. Recently, however, a novel radiation survey meter that uses a plastic-scintillation sensor has been developed. Although manufacturers’ catalog data are available for these survey meters, there have been no user reports on performance. In addition, the performance of commercial plastic-scintillation survey meters has not been evaluated. In this study, we experimentally compared the performance of a plastic-scintillation survey meter with that of a GM survey meter. The results show that the two instruments performed very similarly in most respects. The GM survey meter exhibited count losses when the radiation count rate was high, whereas the plastic-scintillation survey meter remained accurate under such circumstances, with almost no count loss at high radiation rates. For measurements at background rates (i.e., low counting rates), the counting rates of the plastic-scintillation and GM survey meters were similar. Therefore, an advantage of plastic-scintillation survey meters is that they are less affected by count loss than GM survey meters. We conclude that the plastic-scintillation survey meter is a useful β-ray measuring/monitoring instrument.

## 1. Introduction

Survey meters have long played important roles in radiation measurement. In assessments of surface contamination, Geiger–Müller (GM) radiation survey meters have most commonly been employed for β-ray measurements. The Fukushima nuclear accident of 2011 released radioactive materials such as Sr-90, I-131, Cs-134, and Cs-137 into the environment [1,2]. Subsequently, measurements of the external and internal radiation doses to Fukushima prefectural citizens, local soils, and the environment were made by several researchers [3]. At that time, the need for survey meters that monitored environmental radiation and surface radiation contamination increased. Therefore, many types of survey meters were developed/used by various companies [4].

Radiation monitoring/measurement of β-rays (including electron beams) is important in the fields of environmental radiation and radiation medicine [5,6].

**Figure 1:**
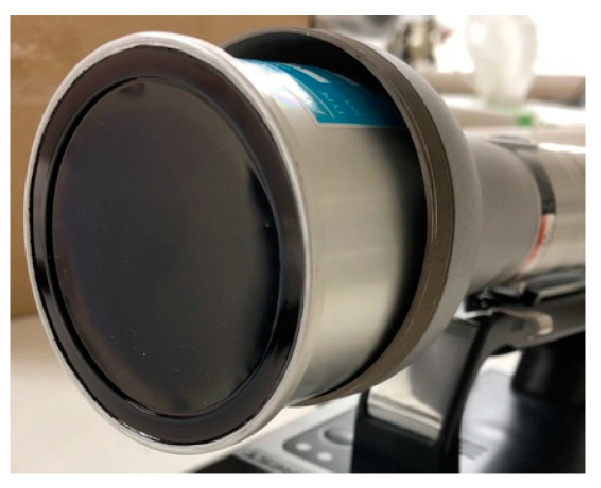
Thin Mica (the incident window of the GM survey meter).

GM survey meters have long been used to measure β-rays [7,8,9,10,11]. They are often used to measure nuclides that emit γ-rays and β-rays (i.e., in mixed fields), such as Cs-137 and I-131. These meters use thin processed mica sheets (Figure 1) for the incident window required for β-ray detection. Given the penetration ability of beta rays, the minimum thicknesses available for mica sheets pose a lower threshold for beta detection. Survey meters in which the radiation sensor is a plastic scintillator can represent a viable alternative. Plastic-scintillation survey meters in which the radiation sensor is a plastic scintillator have thus been developed.

Conventional radiation-measuring instruments such as GM survey meters have been described in many papers [12,13,14,15,16] and several performance reports have appeared [17,18,19]. In contrast, although catalog data on plastic-scintillation survey meters are available [20,21], no user reports on performance for β-rays have appeared. In addition, although papers on plastic scintillators per se have appeared [22,23,24,25], as well as reports of the use of plastic scintillators in various experimental equipment [26], no paper has yet evaluated the performance of a plastic scintillator incorporated into a commercial survey meter. Therefore, it was important to evaluate the basic performance of this type of survey meter experimentally.

## 2. Instruments and Methods

**Figure 2:**
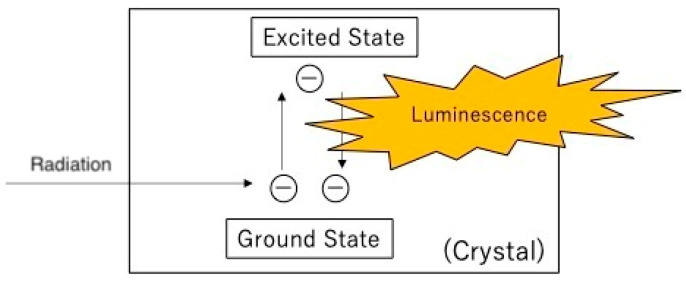
Measurement principle of the plastic scintillator.

Plastic-scintillation survey meters use a solid plastic scintillator as the radiation sensor. The measurement principle is shown in Figure 2. Radiation entering the plastic scintillator excites electrons in the scintillator, which emit light as they return to the ground state. The light is then collected by a photomultiplier, where it is converted to photoelectrons at the photocathode and these are multiplied to produce the output pulse. A plastic scintillator is characterized by a low effective atomic number, which renders it suitable for the measurement of charged particles, including β-rays. The luminescence and decay times are shorter than those of inorganic scintillators. The plastic-scintillation sensor is sturdy. In GM survey meters, radiation measurement is based on ionization of gas in the GM chamber when radiation enters. GM detectors are not sturdy, because of the thin mica window used. In this respect, the plastic-scintillation sensor can offer a more robust solution.

### 2.1. Devices under Test

**Figure 3:**
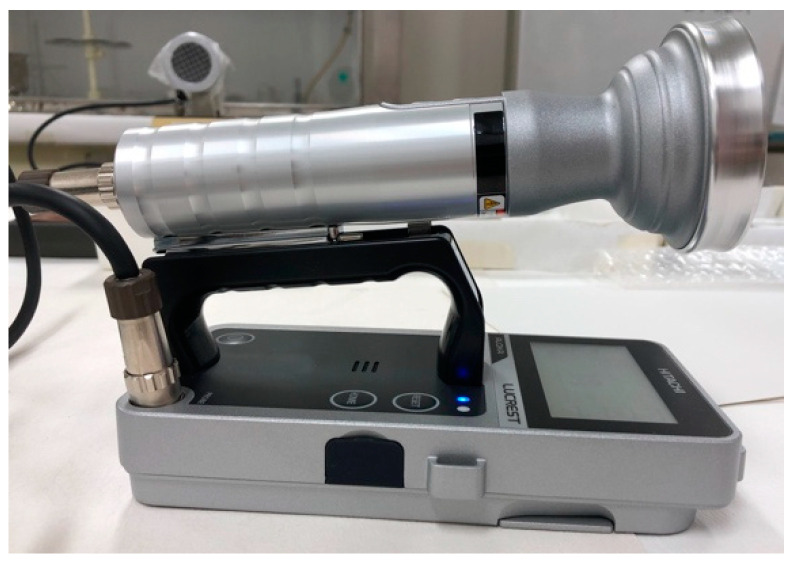
The plastic-scintillation survey meter TCS-1319H.

**Figure 4:**
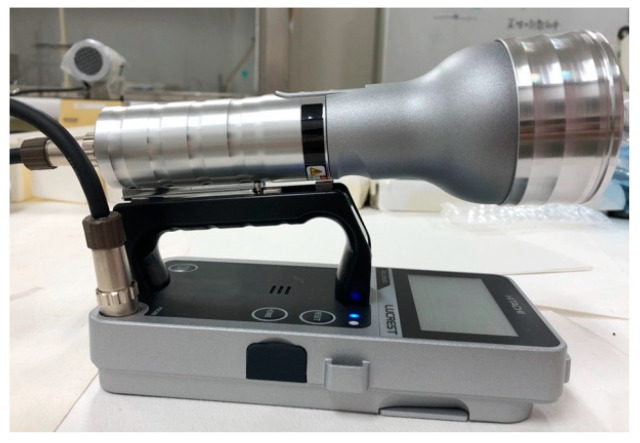
The GM survey meter TGS-1146.

Two types of instruments were used to detect β-rays: a plastic-scintillation survey meter (the LUCREST Rugged Survey Meter TCS-1319H (Figure 3) sold by Nippon Raytec [20]) and a GM survey meter (the TGS-1146 (Figure 4), also sold by Nippon Raytec [21]). Both types of survey meter exhibit relative reference errors within ±25% in catalog data. We used two (Nos. 1 and 2) of each type of instrument, thus four survey meters were used in total.

Note that all measurements in this study were made in scaler mode. Conventionally, the count rate (count per minute [cpm]) mode is used to measure radiation when using survey meters. However, this mode has the disadvantages that time constants must be set and that measurement read-out errors (i.e., time-constant influence) are not uncommon. All of the survey meters used in this study offer scaler mode, which eliminates the need for time-constant settings and reduces reading errors. Therefore, we chose to use scaler mode.

### 2.2. Measurement Setup

**Figure 5:**
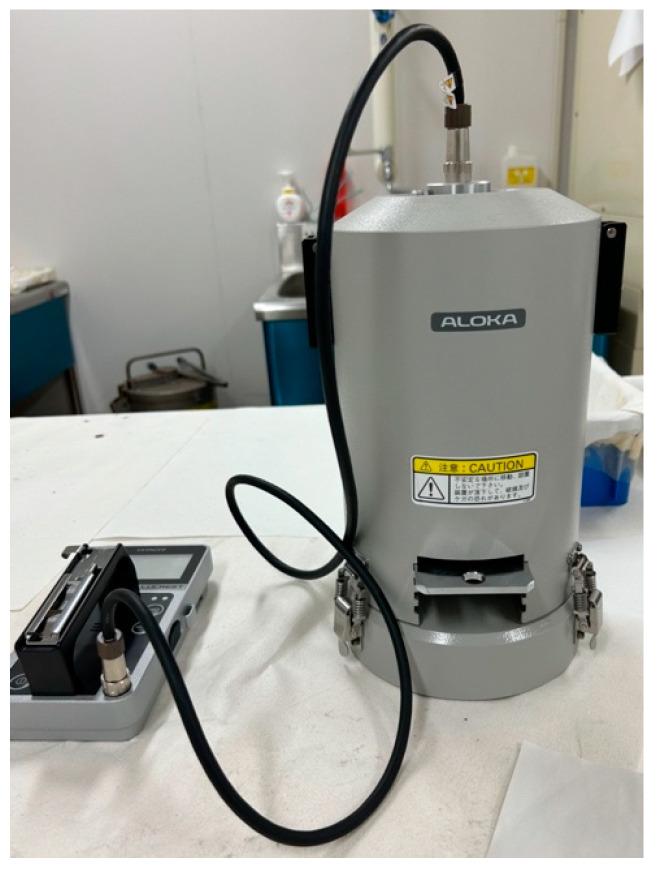
Measurement distance 1.45 cm.

**Figure 6:**
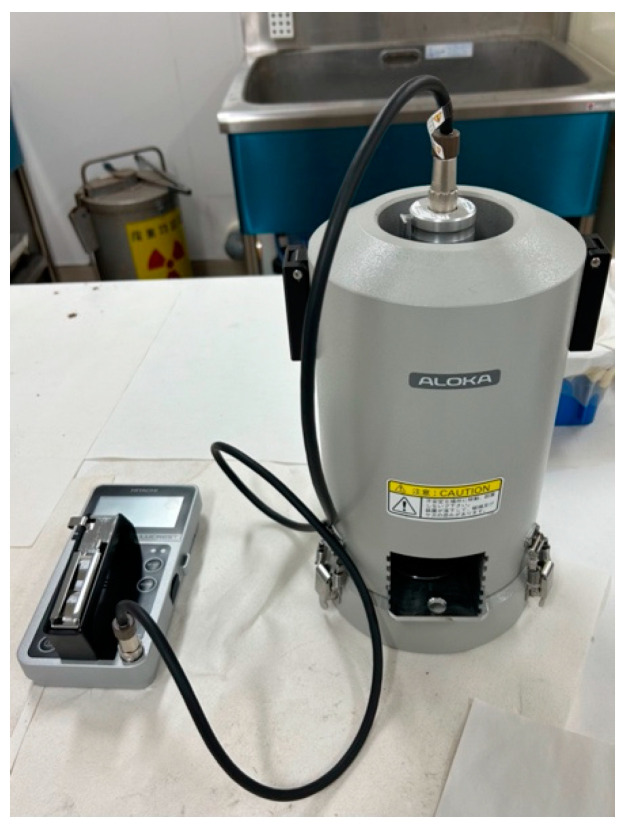
Measurement distance 4.45 cm.

**Figure 7:**
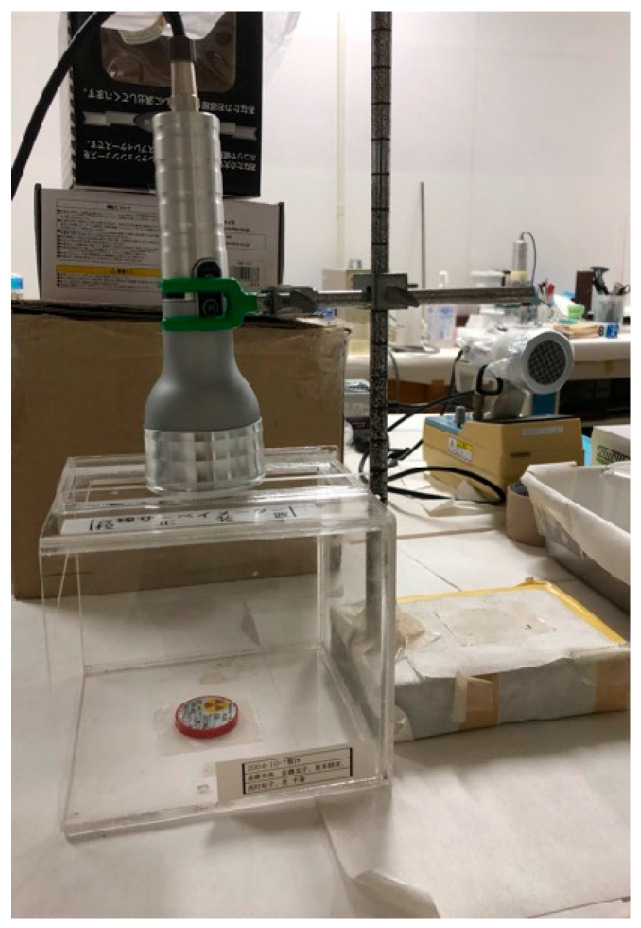
Measurement distance 16 cm.

Counts of the beta radiation of sealed Sr-90 and Cl-36 sources were measured in scaler mode for 1 min (i.e., counts per minute) 10 times using the two types of instruments described in Section 2.1; averages were then obtained as indices of the measured values. We performed the experiment five times for each source. When comparing the values obtained for the sources, the background radiation (BG) was subtracted. The radioactivity values of the Sr-90 and Cl-36 sources were 73.5 kBq and 850 Bq, respectively. The distances from the bottom of the source to the surface of the detector instrument were 1.45 cm, 4.45 cm, and 16 cm. A measuring table (PS-202E) from Nippon Raytec was used for the experiments at 1.45 cm and 4.45 cm. For the experiments at 16 cm, a fixture was used instead of the measuring table to ensure geometrically identical conditions. Figure 5, Figure 6 and Figure 7 show the arrangements used for the measurements.

### 2.3. Measurement Setup (Using an Absorber)

When Sr-90 decays, it emits β-rays up to an endpoint energy of 0.546 MeV. Y-90, a daughter nuclide produced by such decay, further decays and emits β-rays up to an endpoint energy of 2.280 MeV (Table 1). Therefore, when measuring β-rays from a Sr-90 source, two types of β-rays with very different endpoint energies are detected simultaneously. Therefore, we also conducted experiments in which the radiation up to an endpoint energy of 0.546 MeV was shielded. An aluminum absorber plate with an outer diameter of 50 mm and an areal density of 217 mg/cm^2^ was used for shielding. The measurements were performed with the plate on top of the source. The absorber selectively shielded β-rays with energies below the 0.546 MeV Sr-90 endpoint energy. The other measurement conditions were the same as those without shielding. We performed the experiment 10 times each for 1.45, 4.45, and 16 cm.

### 2.4. Resolving-Time Measurements

In this study, the resolving times of the count-rate measurements of a plastic-scintillation survey meter and a GM survey meter (one of each) were also measured.

**Figure 8:**
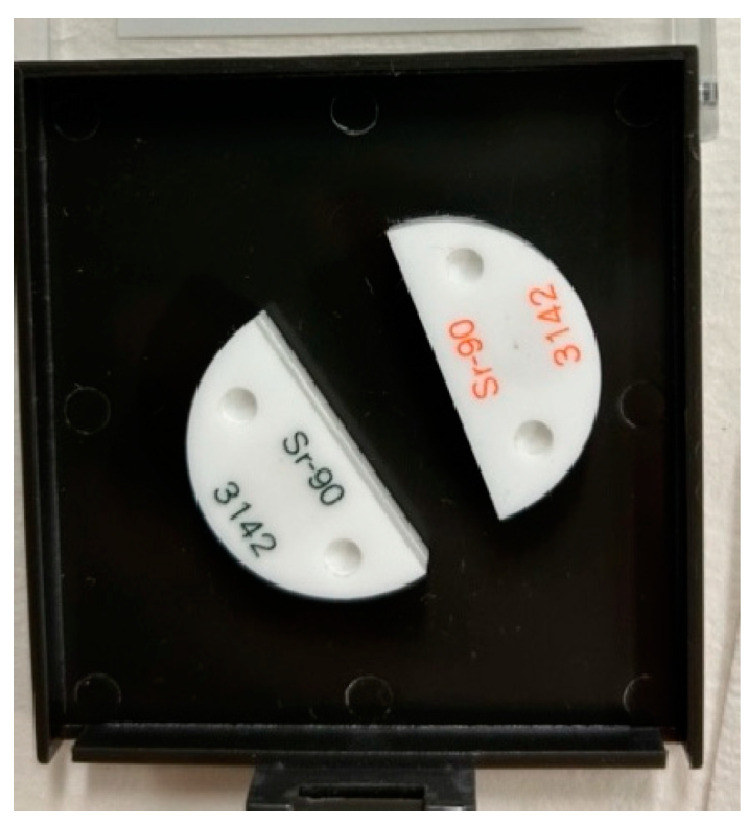
The Sr-90 nuclide source used in the two-source method.

The Sr-90 source shown in Figure 8 was used when employing the two-source method for resolving-time measurements; it consists of two half-moon-shaped sealed sources that can be placed together when combined measurements are required. One source bears source information in orange characters and the other has such information in green characters. Let n_12_ be the count of the two sources measured coincidently, n_1_ the count of the orange source, n_2_ the count of the green source, and n_b_ the background. Then, the resolving time τ can be expressed by the following equation [27]:

τ = (n_1_ + n_2_ − n_12_ − n_b_)/(n_12_^2^ − n_1_^2^ − n_2_^2^)

Counts per minute were measured for four scenarios (both sources at the same time, orange source only, green source only, and background) 10 times each and obtained the average of each. After the measurements, the resolving time (s) was calculated. We performed the experiment five times for the plastic-scintillation survey meters and three times for the GM survey meters, respectively, and the averages were estimated. Two of the four survey meters described in Section 2.1 were used, one of each type. The arrangement during measurements was the same as that for measurements at 4.45 cm described in Section 2.2.

## 3. Results

### 3.1. Measurements of β-Ray-Emitting Nuclides

**Figure 9:**
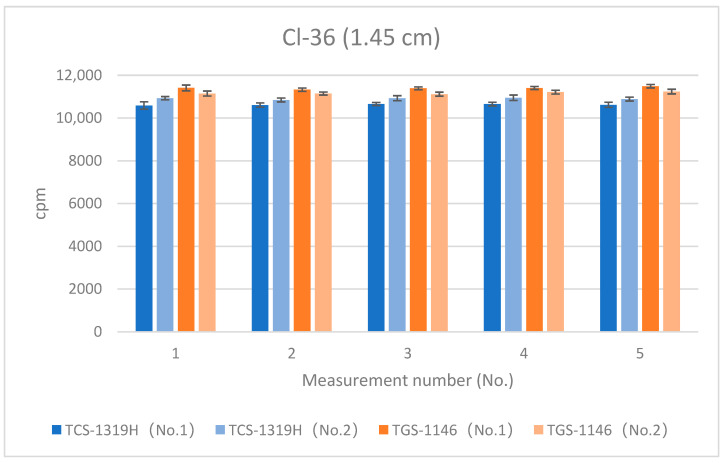
Measurement results for Cl-36 (1.45 cm).

**Figure 10:**
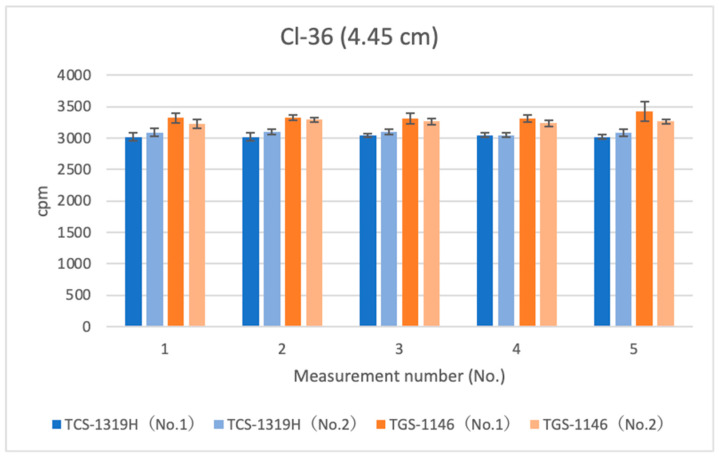
Measurement results for Cl-36 (4.45 cm).

**Figure 11:**
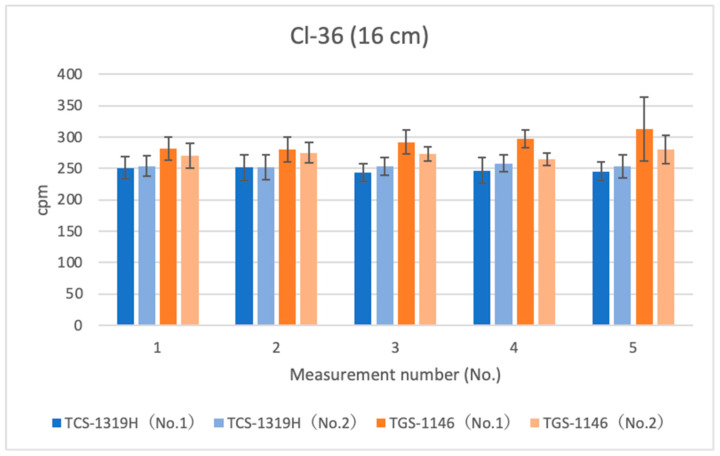
Measurement results for Cl-36 (16 cm).

Figure 9, Figure 10 and Figure 11 show the results for Cl-36 at each distance. Error bars in the graphs indicate standard deviations. For the first plastic-scintillation survey meter (No. 1), the averaged measured values (cpm) at distances of 1.45 cm, 4.45 cm, and 16 cm were 10,624.86, 3027.10, and 247.72 cpm, respectively. For the second plastic-scintillation survey meter (No. 2), the values were 10,908.88, 3083.40, and 253.82 cpm, respectively. For the first GM survey meter (No. 1), the measured values were 11,405.16, 3336.96, and 292.50 cpm, respectively. For the second GM survey meter (No. 2), the values were 11,172.38, 3256.32, and 272.58 cpm, respectively. Compared with the GM survey meters, the differences in the sensitivities of the plastic-scintillation survey meters were −4.62%, −7.32%, and −11.30% at 1.45 cm, 4.45 cm, and 16 cm, respectively (Table 2). According to the catalog data, both types of instruments exhibit relative reference errors within ±25%. In other words, all measurement disparities were within the relative ranges of reference error. At all distances, the GM survey meters yielded slightly higher values. However, the degree of error was not an issue. During measurements of Cl-36, the performances of each survey meter were approximately equivalent.

TGS No. 1 measurement No. 5 differs from the others; it also has a larger error (Figure 11). Although we are unsure of the reason for this difference, it might be due to measurement error.

**Figure 12:**
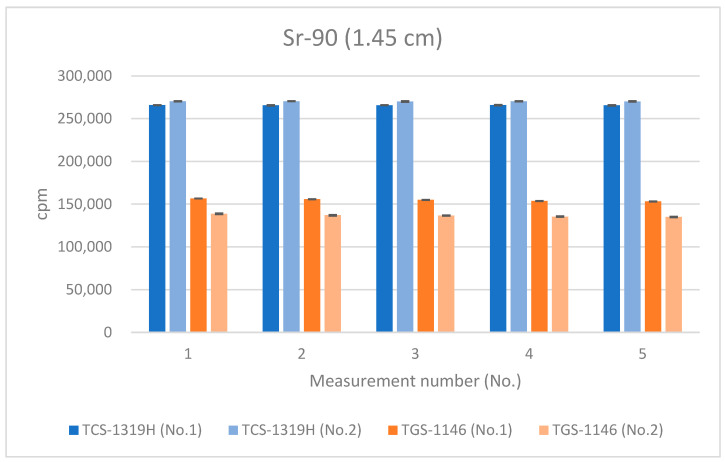
Measurement results for Sr-90 at 1.45 cm distance.

**Figure 13:**
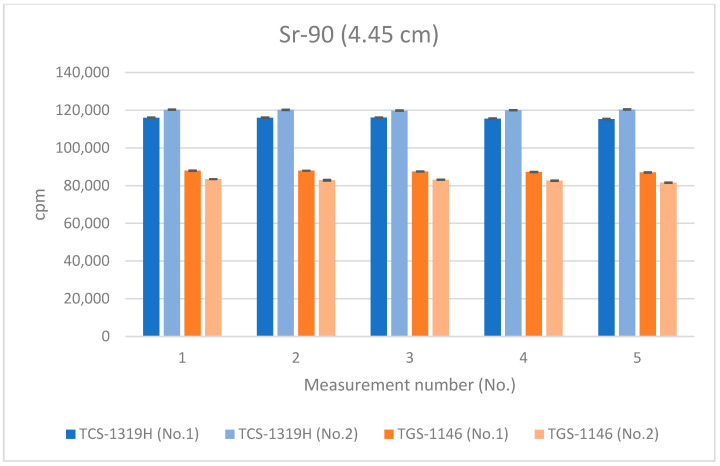
Measurement results for Sr-90 at 4.45 cm distance.

**Figure 14:**
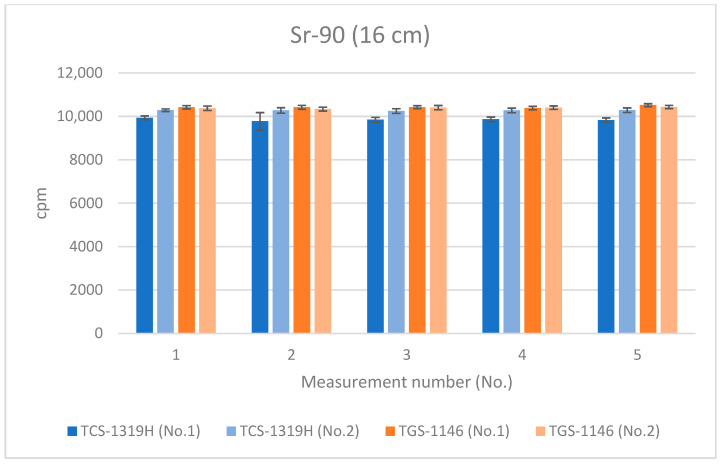
Measurement results for Sr-90 at 16 cm distance.

Figure 12, Figure 13 and Figure 14 show the results for Sr-90 at each distance. All error bars in the graphs indicate standard deviations (N = 10). For the first plastic-scintillation survey meter (No. 1), the averaged measured values at distances of 1.45 cm, 4.45 cm, and 16 cm were 265,731.34, 115,793.44, and 9849.54 cpm, respectively. For the second plastic-scintillation survey meter (No. 2), the values were 270,300.12, 120,131.38, and 10,275.68 cpm, respectively. For the first GM survey meter (No. 1), the measured values were 154,847.78, 87,535.98, and 10,434.40 cpm, respectively. For the second GM survey meter (No. 2), the values were 136,554.34, 82,743.12, and 10,391.46 cpm, respectively. The disparities in the counting rates of the plastic-scintillation survey meters compared to the GM survey meters were 83.95%, 38.55%, and −3.36% at 1.45 cm, 4.45 cm, and 16 cm, respectively (Table 3). The values measured by the two types of meter were closest when the distance was 16 cm. However, at shorter distances, the plastic-scintillation survey meter values were much higher than those of the GM survey meters, with a difference exceeding the relative reference errors. The cause of this result will be investigated and clarified later in the text. 

### 3.2. Measurements of β-Ray-Emitting Nuclides Using an Absorber

**Figure 15:**
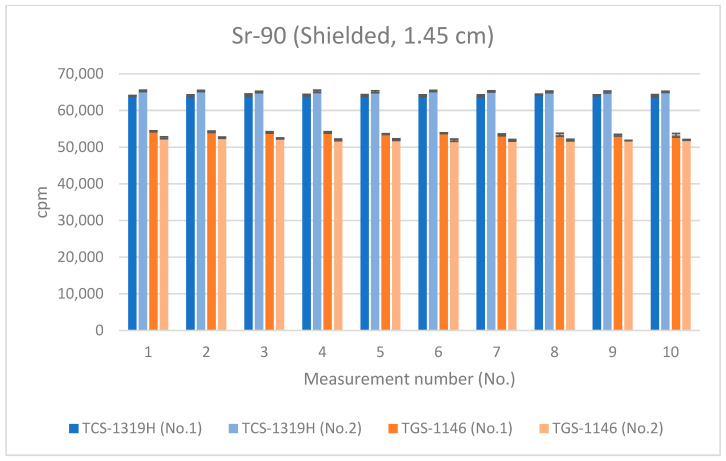
Measurement results for Sr-90 at 1.45 cm distance (using an absorbing plate).

**Figure 16:**
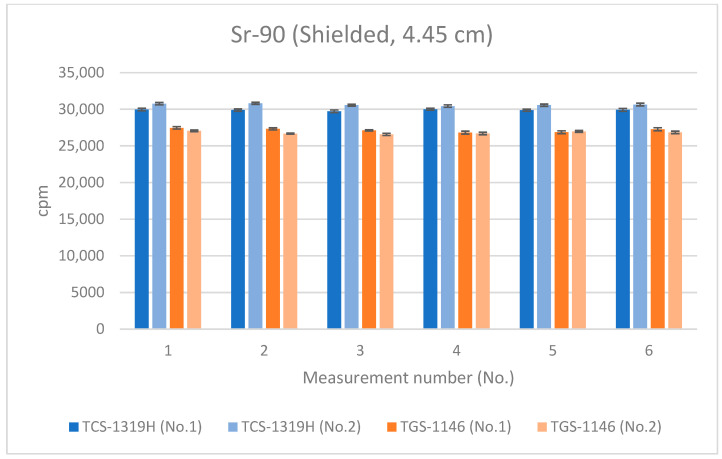
Measurement results for Sr-90 at 4.45 cm distance (using an absorbing plate).

**Figure 17:**
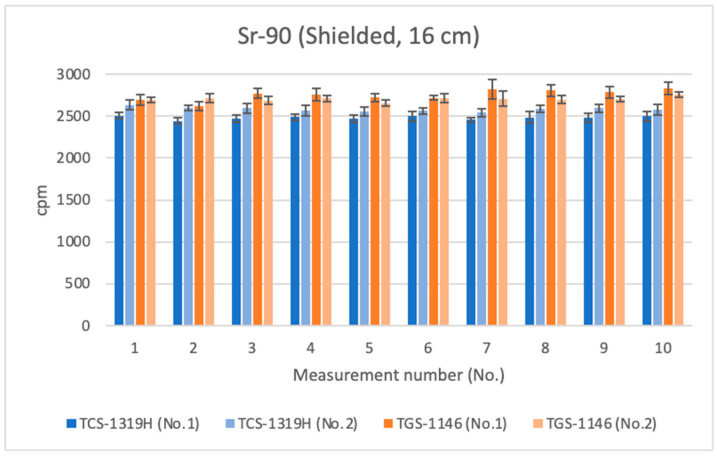
Measurement results for Sr-90 at 16 cm distance (using an absorbing plate).

Next, measurements at the distances detailed in Section 3.1 were repeated with the absorber placed on top of the Sr-90 source; the results are shown in Figure 15, Figure 16 and Figure 17. All error bars in the graphs indicate standard deviations (N = 10). For the first plastic-scintillation survey meter (No. 1), the averaged measured values for Sr-90 at distances of 1.45 cm, 4.45 cm, and 16 cm were 64,107.60, 29,897.48, and 2480.95 cpm, respectively. For the second plastic-scintillation survey meter (No. 2), the values were 65,190.40, 30,631.42, and 2581.94 cpm, respectively. For the first GM survey meter (No. 1), the measured values were 53,730.13, 27,145.73, and 2753.90 cpm, respectively. For the second GM survey meter (No. 2), the values were 52,113.02, 26,804.48, and 2705.19 cpm, respectively (Table 4). The disparities in the counting rates of the plastic-scintillation survey meters compared to the GM survey meters were 22.16%, 12.19%, and −7.26% at 1.45 cm, 4.45 cm, and 16 cm, respectively. As in Section 3.1 above, when the detection distance was short, the values measured by the plastic-scintillation survey meters were larger than those measured by the GM survey meters. However, the disparities were much lower than when the absorber was not used. 

### 3.3. Resolving-Time Measurements

The resolving times of both types of survey meters are based on the measured values of the No. 1 instruments. The average resolving time of the GM survey meter was 292 μs for the three sets. That of the plastic-scintillation survey meter was less than 1 μs, on average, for the five sets; thus, very small compared to that of the GM survey meter. According to the TGS-1146 catalog, the decomposition time is approximately 250 μs. Thus, this can be considered a reasonable result, at least for the GM survey meter.

## 4. Discussion

In the field of radiation medicine, exposure of patients and staff to radiation is an issue of great importance [28,29,30,31,32,33,34,35,36,37]. To date, many studies have evaluated radiation doses and protection from exposure [38,39,40,41,42,43]. Our laboratory has also published many research papers on patient radiation measurements, occupational exposure evaluation, and how to reduce radiation doses [44,45,46,47]. Radiation exposure in nuclear medicine using radioactive materials (radiopharmaceuticals) is another important problem [48,49]. Radiation monitoring using a survey meter is often performed in rooms where nuclear medicine and X-ray medicine are applied. Radiation survey meters are important, not only in monitoring nuclear facilities but also in medical settings. Thus, we investigated the performance of a new radiation survey meter with a plastic-scintillation sensor because there have been no user reports regarding the performance of plastic-scintillation survey meters.

To the best of our knowledge, this is the first paper to offer a basic performance evaluation of a plastic-scintillation survey meter.

When Cl-36 and Sr-90 sources were measured without an absorber, the values provided by the GM survey meters were lower only for the Sr-90 source.

When Cl-36 was measured, the GM survey meters had slightly higher readings than the plastic-scintillation survey meters (Figure 12, Figure 13 and Figure 14), likely because of measurement error. Another reason may be that as the distance between the source and detector increased, the β-ray energy was affected by air-induced attenuation. For the GM survey meters, the incident β-ray ionizes the organic gas in the GM tube; the resulting wave height is independent of the incident energy of the β-ray. In contrast, the wave height of the plastic scintillation survey meters depends on the energy of the β-ray that reaches the scintillator. Therefore, the plastic-scintillation survey meter may have produced lower readings than the GM survey meter. This suggests that the difference in sensitivity (cpm) between the two models increased according to the distance between the source and detector.

According to the catalog data, the measurement ranges are 0–300 kcpm for the plastic-scintillation survey meter (TCS-1319H) and 0–100 kcpm for the GM survey meter (TGS-1146). This means that the upper limit of the measurement range was exceeded only when the Sr-90 source was measured using the GM survey meter without the absorber.

Furthermore, if the radioactivity of the source is A, the number of counts per second is N, the fraction of solid angle covered by the detector is λ_1_, and the counting efficiency of the device is λ_2_, then Equation (1) holds:

N = λ_1_λ_2_*A* (1)

By transforming this equation, the counting efficiency of the device, λ_2_, can be expressed using Equation (2):

λ_2_ = N/λ_1_*A* (2)

**Figure 18:**
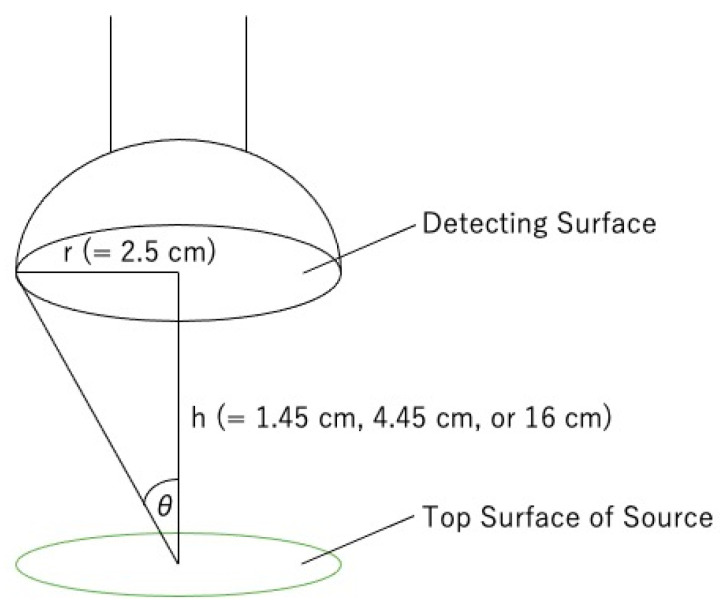
The measurement conditions.

As noted in Section 2.2, the radioactivity A is 850 Bq for the Cl-36 source and 73.5 kBq for the Sr-90 source. The fraction of solid angle covered by the detector λ_1_ can be expressed by Equations (3) and (4) when the distance between the source and the detector is h (1.45 cm, 4.45 cm, or 16 cm) and the radius of the detector surface is r (2.5 cm for both the plastic-scintillation survey meters and the GM survey meters) as shown in Figure 18.

cos *θ* = h/√(*r*^2^ + h^2^) (3)

λ_1_ = (1 − cos *θ*)/2 (4)

Substituting Equation (4) into Equation (2) yields Equation (5):

λ_2_ = 2N/{A(1 − cos *θ*)} (5)

**Figure 19:**
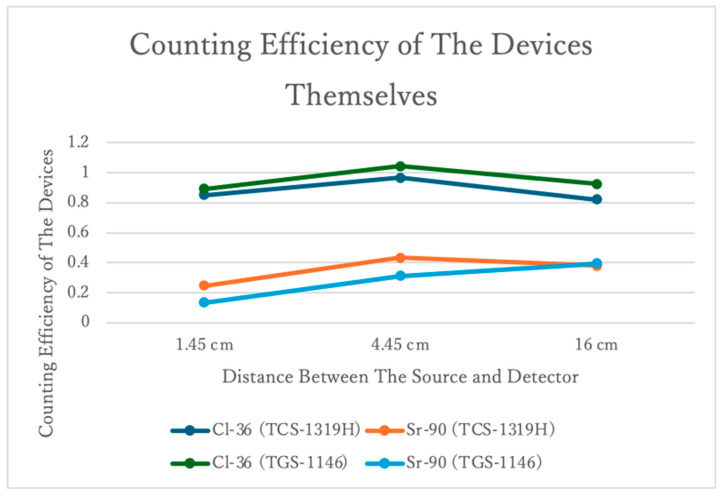
The counting efficiencies of the devices per se.

Insertion of the corresponding values for N, A, r, and h into Equations (3) and (5), allows the graph for λ_2_ (Figure 19) to be drawn.

The graph shows that the counting efficiencies of both the plastic-scintillation survey meters and the GM survey meters are smaller during Sr-90 measurements compared to Cl-36 measurements. Both instrument types may not be able to adequately measure the high-energy β-rays from Y-90. The decreases in the counting efficiencies of the GM survey meters were particularly noticeable as the distance between the meter and the source decreased.

The efficiency slightly exceeded 1.0 when Cl-36 was measured at 4.45 cm (Figure 19), but since it is theoretically impossible for efficiency to exceed 1, this result may reflect an error.

**Figure 20:**
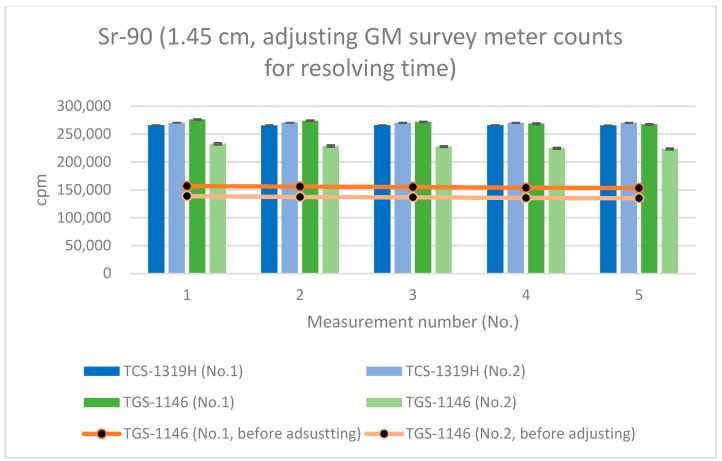
Measurement results for Sr-90 at 1.45 cm distance (with adjustment of the GM survey meter counts for the resolving time).

**Figure 21:**
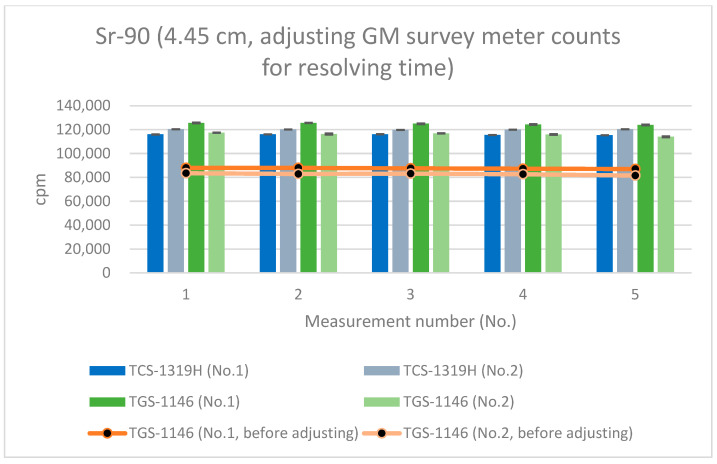
Measurement results for Sr-90 at 4.45 cm distance (with adjustment of the GM survey meter counts for the resolving time).

**Figure 22:**
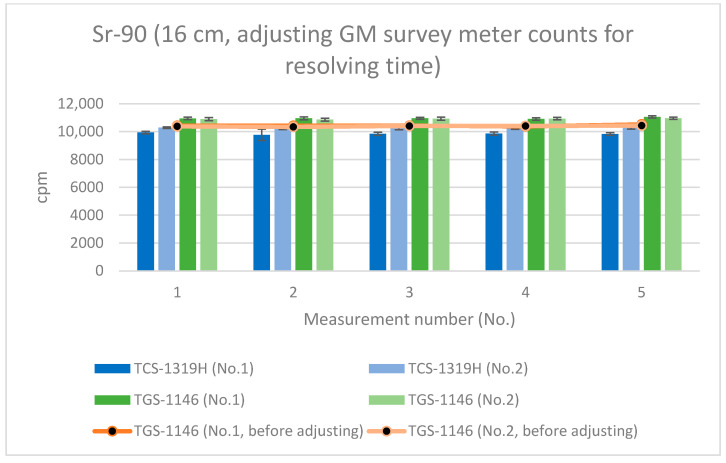
Measurement results for Sr-90 at 16 cm distance (with adjustment of the GM survey meter counts for the resolving time).

As mentioned in Section 3.3, the average resolving time of the GM survey meter was 292 μs, whereas that of the plastic-scintillation survey meter was less than 1 μs. If the counts for the GM survey meters are adjusted for the counting losses (thus, by applying count loss corrections, i.e., using Equation (6)), Figure 15, Figure 16 and Figure 17 become transformed into Figure 20, Figure 21 and Figure 22. Note that Equation (6) is valid when the true counting rate (cpm) is R, the actual counting rate (cpm) is r, and the resolving time is τ:

R/60 = r/60 + (r/60)^2^τ (6)

Equation (6) will essentially provide an approximation that is valid for low counting rates; it may be ineffective when applied to highly radioactive sources.

All error bars in the graphs indicate standard deviations (N = 10). From these results (the graphs of Figure 20, Figure 21 and Figure 22), it can be seen that the counting rates of the plastic-scintillation survey meters and the GM survey meters were comparable after adjusting for the counting losses of the GM survey meters. The disparities in the counting rates of the plastic-scintillation survey meters relative to the GM survey meters became 7.42%, −2.08%, and −8.05% at 1.45 cm, 4.45 cm, and 16 cm, respectively. The results for TGS-1146 (no. 2) remain lower than the others at 1.45 cm. Individual differences between models may have affected this result (they are not sufficiently large to be problematic).

Therefore, the large disparities in the readings at close range only when the Sr-90 source was measured without the absorber were attributable to counting losses caused by the long resolving time associated with high numbers of β-rays and were more pronounced for GM survey meters. Therefore, if using a GM survey meter in a situation in which a very large number of β-rays is detected, it is necessary to ensure that the counting loss is adjusted for via count loss correction. When obtaining measurements using the plastic-scintillation survey meter, the measured values do not change significantly, so no count loss correction is needed. However, in situations where the counts are very high, the influence of count loss may even be present in a plastic scintillator. Therefore, if immediate count rates are required from a high-radiation field, a plastic-scintillation survey meter is recommended. For measurements at background rates (i.e., low counting rates), the counting rates of the plastic-scintillation and GM survey meters were similar.

Furthermore, typically, GM survey meters suffer from degeneration of the GM tube resulting from gas depletion and/or leakage from the detector. Thus, a GM survey meter that has been in use for a long time may exhibit low sensitivity. In a plastic-scintillation survey meter, there is little degeneration of the solid scintillation sensor.

In summary, radiation monitoring is important both in the field of radiation medicine and in atomic power plants [1,3]. Verification of radiation-survey-meter performance is necessary. We evaluated the basic performance of a novel β-ray survey meter that uses a plastic-scintillation sensor. We compared the fundamental characteristics of this meter to those of a GM survey meter, which is the traditional β-ray survey meter. The plastic-scintillation survey meter exhibited roughly the same basic performance as the GM survey meter. However, gas degeneration is not an issue when using a plastic-scintillation sensor. The GM survey meter exhibited counting losses at very high counting rates. Finally, we are of the view that the overall characteristics of the plastic-scintillation survey meter were good.

## 5. Conclusions

In this study, the measurements (counting rates) of a plastic-scintillation survey meter exposed to β-ray-emitting radionuclides were evaluated and compared to those of a Geiger–Muller survey meter. The counting rates for the Cl-36 source were comparable. The values for the Sr-90 source measured using the plastic-scintillation survey meter were higher than those measured using the GM survey meter and increased with increasing counting rate. If the count number is not too high, the plastic-scintillation survey meter is as effective as the GM survey meter. As the count increases to very high values, the count loss of the GM survey meter becomes noticeable. The very short resolving time with almost no count loss is one of the advantages of plastic-scintillation survey meters. Furthermore, the solid plastic-scintillation sensor is sturdy. We conclude that the plastic-scintillation survey meter is a useful β-ray measuring/monitoring instrument.

## Figures and Tables

**Table 1:** The endpoint energy of radiation emitted from the nuclides used in the experiments.

Nuclide	Cl-36	Sr-90	Y-90 (Daughter Nuclide of Sr-90)
Endpoint energy of radiation [MeV]	β − 0.709 (98.1%) β + 0.120 (0.014%) EC (Electron Capture) (1.9%)	β − 0.546 (100%)	β − 2.280 (100%)

**Table 2:** Difference in measured values using plastic-scintillation survey meters and GM survey meters (using Cl-36).

Distance	1.45 cm	4.45 cm	16 cm
The difference in sensitivity of the plastic scintillation survey meter relative to the GM survey meters	−4.62%	−7.32%	−11.3%
Whether the difference is within the relative reference error (≤±25%)	Yes.	Yes.	Yes.

**Table 3:** Difference in measured values using plastic-scintillation survey meters and GM survey meters (using Sr-90).

Distance	1.45 cm	4.45 cm	16 cm
The difference in sensitivity of the plastic scintillation survey meter relative to the GM survey meters	83.9%	38.6%	−3.36%
Whether the difference is within the relative reference error (≤±25%)	No.	No.	Yes.

**Table 4:** Difference in measured values using plastic-scintillation survey meters and GM survey meters (using Sr-90, and absorbing plates).

Distance	1.45 cm	4.45 cm	16 cm
The difference in sensitivity of the plastic scintillation survey meter relative to the GM survey meters	22.2%	12.2%	−7.26%
Whether the difference is within the relative reference error (≤±25%)	Yes.	Yes.	Yes.

## Data Availability

Data are contained within the article.
